# Supplementary material for: Identification of Candidate Chemosensory Gene Families by Head Transcriptomes Analysis in the Mexican Fruit Fly, Anastrepha ludens Loew (Diptera: Tephritidae)
Source: Int J Mol Sci. 2022 Sep 11;23(18):10531. doi: 10.3390/ijms231810531 (PMC9500802; doi:10.3390/ijms231810531)
Supplement: Supplementary file 1 [file ijms-23-10531-s001.zip › ijms-1878782-supplementary.pdf]

Supplementary Information for

Identification of candidate chemosensory gene families by head transcriptomes analysis in the Mexican fruit fly, *Anastrepha ludens* Loew (Diptera: Tephritidae).

Obdulia L. Segura-León [1,*]; Brenda Torres-Huerta [1,*]; Alan Rubén Estrada-Pérez[2]; Juan Cibrián-Tovar[1]; Fidel de la Cruz Hernández-Hernández[3]; José Luis Cruz-Jaramillo[4], José Salvador Meza-Hernández [5]; Fabián Sánchez-Galicia[6]

[1] Entomology and Acarology Program, Colegio de Postgraduados Campus Montecillo, Mexico-Texcoco, km. 36.5, C.P. 56230, State of Mexico, Mexico.
[2] Escuela Superior de Medicina (ESM), Intituto Politécnico Nacional. Casco de Santo Tomas, Miguel Hidalgo, C.P. 11340, Mexico City, Mexico.
[3] Department of Infectomics and Molecular Pathogenesis, Center for Research and Advanced Studies (CINVESTAV-IPN), Mexico City, Mexico.
[4] Bioinformatics and Technologies Department. Solaria Biodata. Col del Valle Centro, Benito Juarez, C.P. 03100, Mexico City, Mexico.
[5] Programa Operativo de Moscas, SENASICA-SADER/IICA, Metapa de Dominguez, Chiapas, Mexico.
[6] Servicio Nacional de Sanidad Inocuidad y Calidad Agroalimentaria, Secretaría de Agricultura y Desarrollo Rural.

*** Correspondence:**
sleon@colpos.mx (O.L.S.-L.)
torres.brenda@colpos.mx (B.T.-H.)

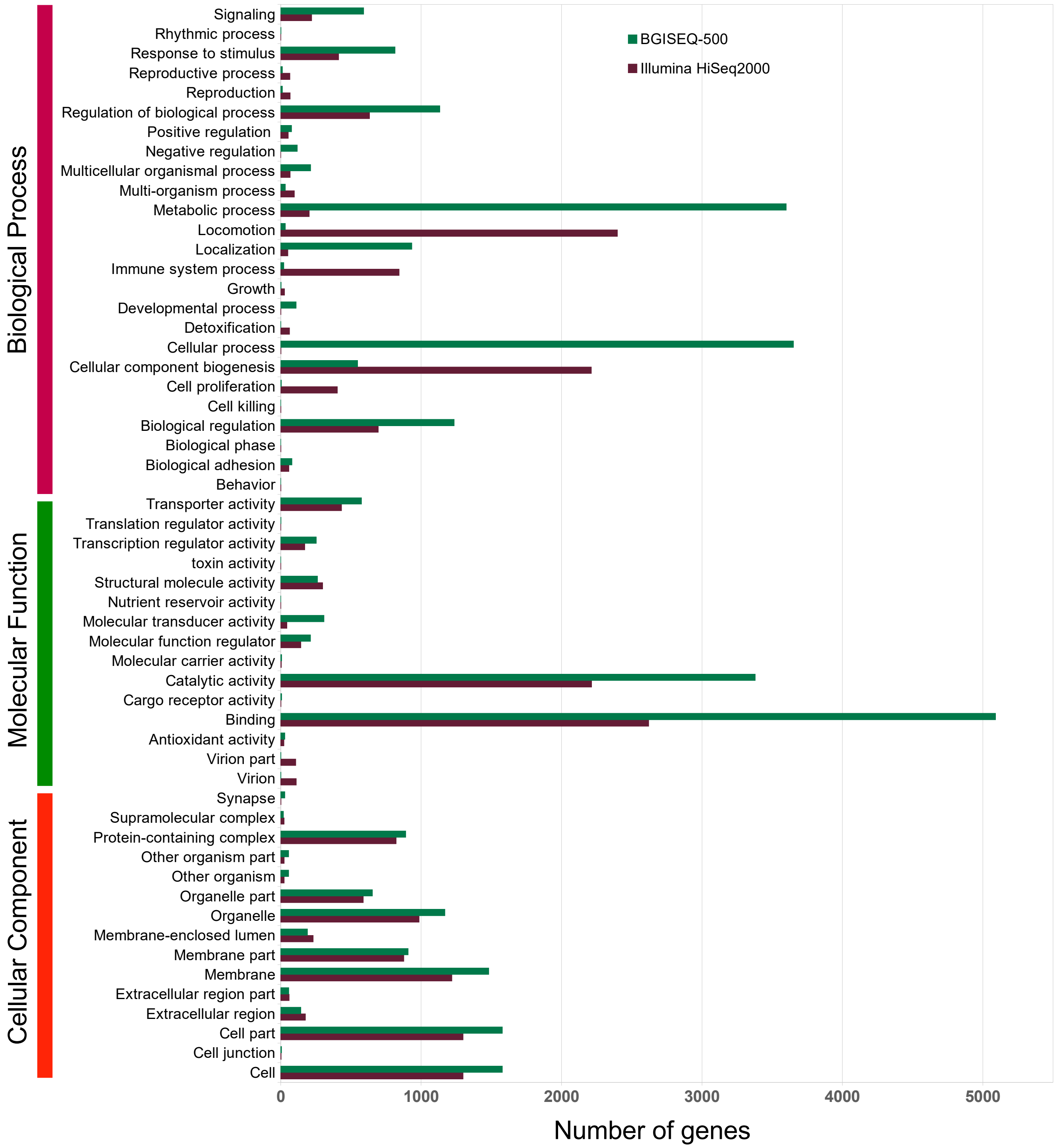

Figure S1. GO term distribution of the 5, 462 *A. ludens* unigenes obtained with the Illumina HiSeq2000 platform and the 7,383 unigenes obtained with the BGISEQ-500 platform classified into the three Gene Ontology (GO) categories of biological process, cellular component and molecular function.

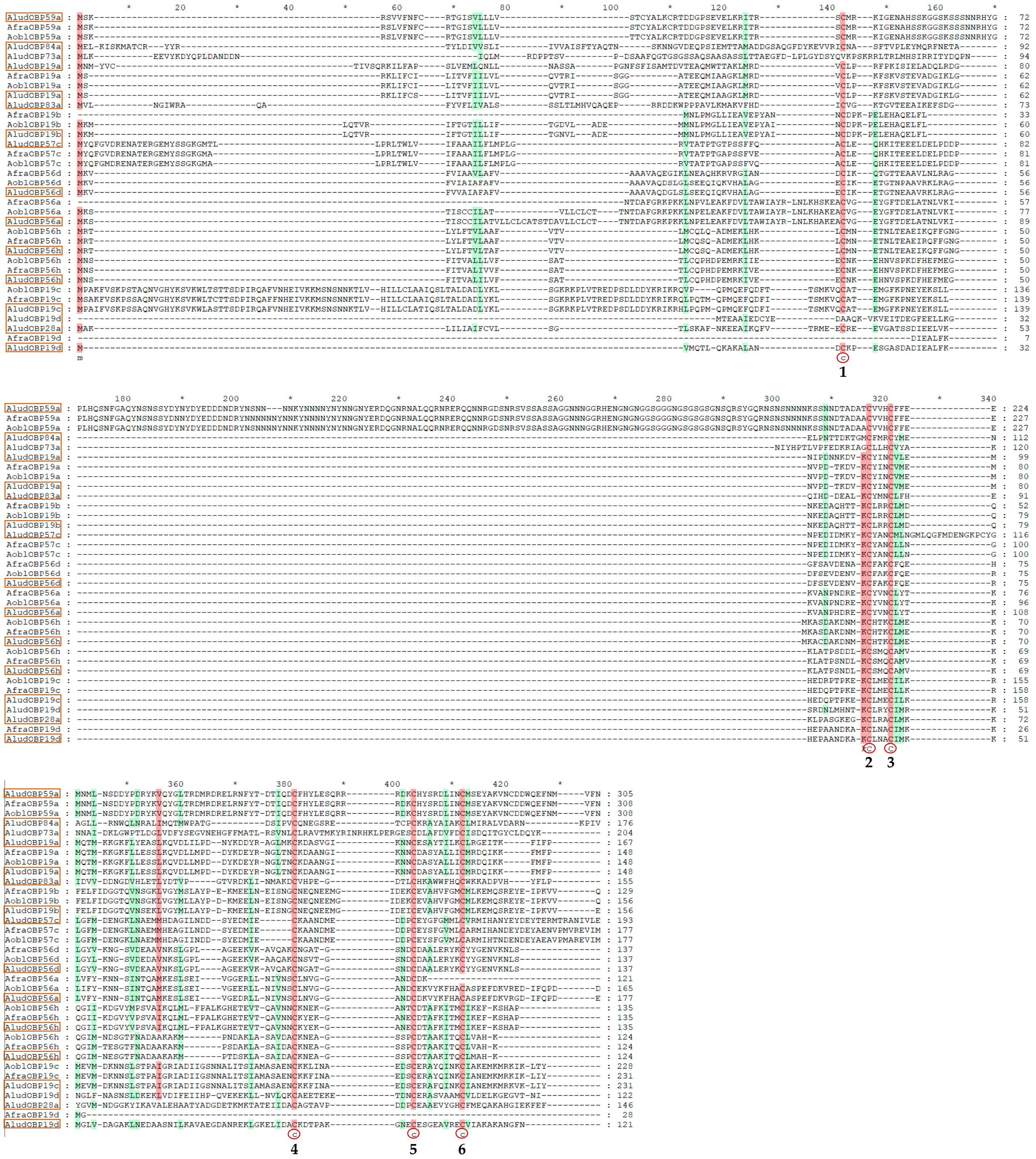

Figure S2. Multiple sequence alignment of *A. ludens* classics OBPs. Amino acid sequences were aligned by Clustal Omega and edited using Genodoc. Color boxes show conserved residues.

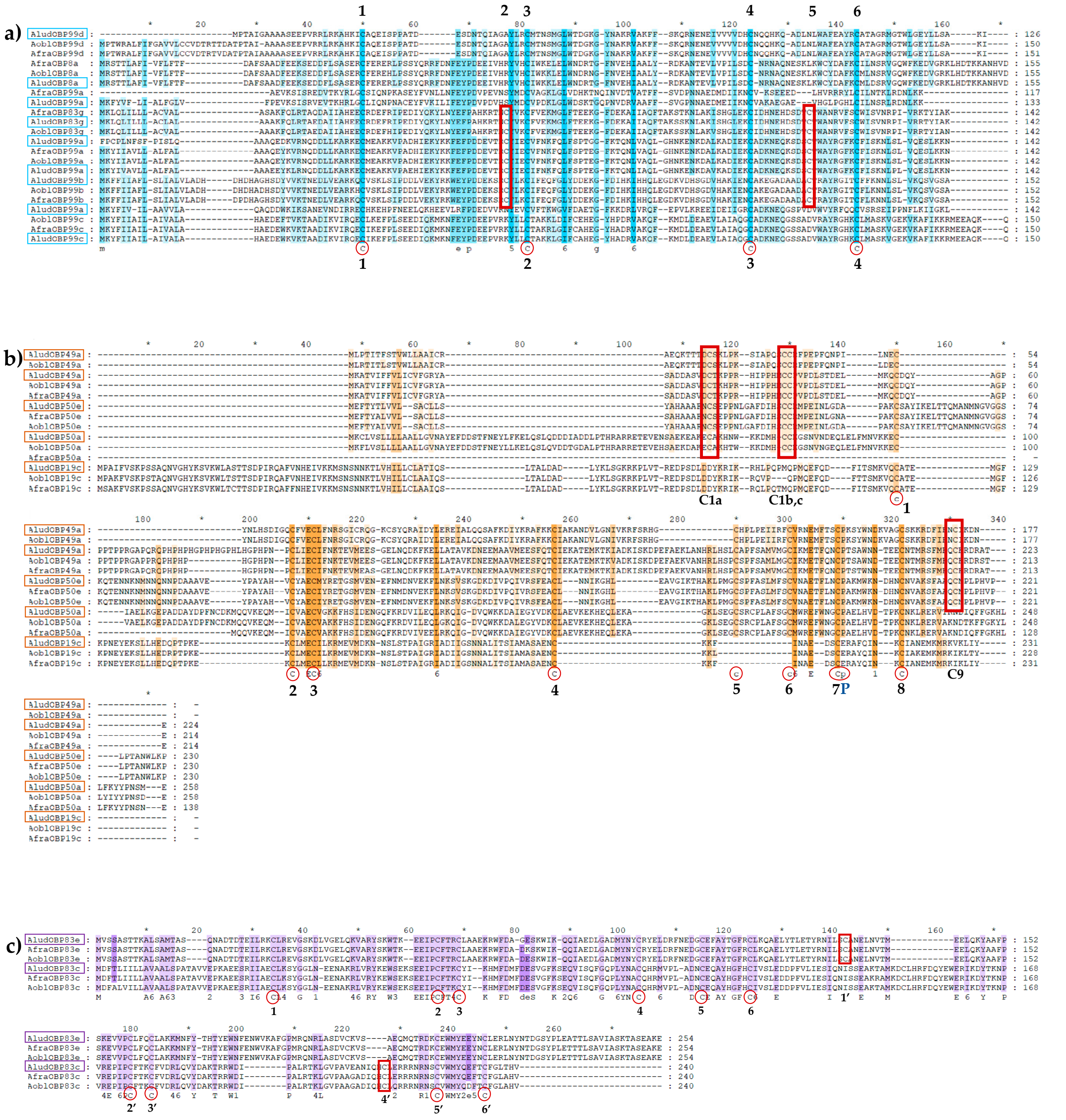

Figure S3. Multiple sequence alignment of *A. ludens* Minus-C (a), Plus-C (b), and Dimers (c) OBPs. Amino acid sequences were aligned by Clustal Omega and edited using Genodoc. Color boxes show conserved residues.

Table S1. Resume Illumina HiSeq2000 sequencing and assembly of of five *A. ludens* libraries

Libraries	RIN values	Media length (bp)	Clean reads	GC%	Q20	Q30	Tasa duplicación
1	5.7	75	6,269,838	27.60	94.71	86.69	86.69
2	5.7	75	5,923,008	39.84	90.36	79.50	79.50
3	5.9	75	8,124,304	39.23	91.23	81.41	81.41
4	6	75	4,602,654	39.93	95.04	87.00	87.00
5	6	75	9,213,200	45.18	90.54	80.61	80.61

	Transcritos	Unigenes	LM (pb)	N30	N50	GC%
**Ensamble *de novo***	24,368	21,103	359	-	880	38.56

Table S2. Resume BGISEQ-500 sequencing and assembly of of six *A. ludens* libraries

Libraries	RIN values	Media length (bp)	Clean reads	GC%	Q20	Q30	Tasa duplicación
MCLL-R1	6.4	99pb	45,865,416	39.74	97.46%	90.03%	2.06
MCLL-R2	6.2	99pb	48,235,940	39.74	97.62%	90.48%	2.63
MCLL-R3	6.2	99pb	48,353,610	40.18	97.75%	90.96%	2.98
MSLL-R1	6	99pb	48,346,156	39.30	97.79%	91.02%	2.86
MSLL-R2	6	99pb	48,657,144	40.07	97.63%	90.53%	3.22
MSLL-R3	6.2	99pb	48,201,102	39.14	97.73%	90.89%	3.37

	Transcritos	Unigenes	LM (pb)	N30	N50	GC%
**Ensamble *de novo***	269,924	158,693	358	2,619	1,245	36.85

Table S3. Best Blastp match of *A. ludens* candidate OBPs

Gene	ORF (aa)	Type ORF	SP	Group	Best Blastp Match				
					Name	Acc. No.	Species	Evalue	Identity (%)
AludOBP8a	156	C	1-22	Minus-C	OBP8a	A0A1D8QLL7	*Anastrepha fraterculus*	0	98.07
AludOBP19a1	149	5p	No	Classic	OBP19a	A0A6M9TZQ0	*Zeugodacus tau*	2.50E-85	82.99
AludOBP19a2	149	C	1-27	Classic	OBP19a	A0A1D8QLH6	*Anastrepha obliqua*	1.51E-104	99.32
AludOBP19b	157	C	1-25	Classic	OBP19b	A0A1D8QLJ0	*Anastrepha obliqua*	6.03E-109	97.44
AludOBP19c	232	C	No	Classic	OBP19c	A0A1D8QLL9	*Anastrepha fraterculus*	8.09E-165	95.24
AludOBP19d1	122	C	No	Classic	OBP19d2	A0A6M9TZR3	*Zeugodacus tau*	8.07E-34	50.43
AludOBP19d2	110	I	No	Classic	OBP19d2	A0A3G2LEG3	*Bactrocera minax*	0	50.48
AludOBP28a	147	C	1-21	Classic	OBP28a	A0A3G2LEG5	*Bactrocera minax*	3.14E-70	69.39
AludOBP49a1	232	5p	1-28	Plus-C	OBP49a	A0A1D8QLL5	*Anastrepha fraterculus*	1.64E-152	93.75
AludOBP49a2	184	C	1-26	Plus-C	OBP49a2	A0A1D8QLH9	*Anastrepha obliqua*	6.25E-128	98.31
AludOBP50a	126	I	1-19	Plus-C	OBP50a	A0A1D8QLI9	*Anastrepha obliqua*	0	92.56
AludOBP50e	215	5p	1-21	Plus-C	OBP50e	A0A1D8QLI2	*Anastrepha obliqua*	1.28E-109	98.71
AludOBP56a	178	C	1-25	Classic	OBP56a	A0A1L2JIV6	*Anastrepha obliqua*	1.97E-112	89.83
AludOBP56d	138	C	1-19	Classic	OBP56d1	A0A1D8QLK6	*Anastrepha obliqua*	0	94.83
AludOBP56h1	150	5p	1-37	Classic	OBP56h1	A0A1D8QLM0	*Anastrepha fraterculus*	2.99E-95	97.78
AludOBP56h2	125	C	1-20	Classic	OBP56h2	A0A1D8QLM2	*Anastrepha fraterculus*	7.04E-85	95.97
AludOBP57c	129	C	No	Classic	OBP57c	A0A1D8QLM5	*Anastrepha fraterculus*	3.69E-70	92.86
AludOBP59a	306	C	1-27	Classic	OBP59a	A0A1D8QLK1	*Anastrepha obliqua*	0	98.05
AludOBP73a	267	C	No	Classic	OBP73a	A0A6M9TZR8	*Zeugodacus tau*	3.04E-84	60.49
AludOBP83a	156	C	1-32	Classic	OBP83a	A0A0G2UGI4	*Bactrocera dorsalis*	4.49E-104	90.32
AludOBP83cd	271	5p	No	Dimer	OBP83cd	A0A1D8QLM6	*Anastrepha fraterculus*	5.35E-179	97.92
AludOBP83g	143	C	1-18	Classic	OBP83g	A0A1D8QLM7	*Anastrepha fraterculus*	0	97.52
AludOBP83ef	282	C	1-32	Dimer	OBP83ef	A0A1D8QLK5	*Anastrepha obliqua*	0	99.21
AludOBP84a	177	C	No	Classic	GOBP84a	A0A1D6Y6I5	*Zeugodacus tau*	0	70.19
AludOBP99a1	154	5p	1-29	Minus-C	OBP99a	W8ASY6	*Ceratitis capitata*	6.55E-101	100
AludOBP99a-like	135	I	1-22	Minus-C	OBP99a	A0A0Y0P9P1	*Anastrepha fraterculus*	4.29E-52	70.94
AludOBP99a2	108	C	No	Minus-C	OBP99a	A0A1D8QLK7	*Anastrepha obliqua*	6.99E-75	100
AludOBP99a3	149	5p	1-23	Minus-C	OBP99a	W8BYY3	*Ceratitis capitata*	1.54E-105	99.32
AludOBP99b	173	C	No	Minus-C	OBP99b	A0A1D8QLN0	*Anastrepha fraterculus*	0	98.68
AludOBP99c	102	C	No	Minus-C	OBP99c	A0A1D8QLN2	*Anastrepha fraterculus*	1.05E-69	99.01
AludOBP99d	127	C	No	Minus-C	OBP99d	A0A1D8QLN1	*Anastrepha fraterculus*	0	98.4

ORF: Open Reading Frame, C: complete, 5p: 5prime partial, I: internal partial; SP: signal peptide

Table S4. Best Blastp match of *A. ludens* candidate CSPs.

Gene	ORF (aa)	Type ORF	Best Blastp Match UniprotKB				
			Name	Acc. No.	Species	Evalue	Identity (%)
AludCSP1	129	Complete	CSP1	A0A6M9TZS5	*Zeugodacus tau*	1.24E-076	87.5
AludCSP2	141	Complete	CSP2	AYN70622	*Bactrocera minax*	1E-080	92.13
AludCSP4	126	Complete	CSP4	A0A6M9U045	*Zeugodacus tau*	1.99E-066	88.28
**Gene**	**ORF (aa)**	**Type ORF**	**Best Blastp Match NCBI**				
			Name	Acc. No.	Species	Evalue	Identity (%)
AludCSP3-1	51	3 partial	CSP	QKN21121	*Bactrocera dorsalis*	5e-19	88.37%
AludCSP3-2	149	Complete	uncharacterized	XP011208558	*Bactrocera dorsalis*	3e-73	79.87%

Table S5. The Blastp match of *A. ludens* candidate SNMPs.

Gene	ORF (aa)	Type ORF	Transmembrane helices	Best Blastp Match				
				Name	Acc. No.	Species	E-value	Identity (%)
AludSNMP1b	509	Complete	1: 20-37<br>2: 466-488	SNMP1	W8C9T0	*Ceratitis capitata*	0	90.89
AludSNMP2	630	Complete	1: 79-101<br>2: 595-617	SNMP2	A0A034WBN1	*Bactrocera dorsalis*	0	95.49

Table S6. Best Blastp match of *A. ludens* candidate ORs

Gene	ORF (aa)	Type ORF	TMH	Best Blastp Match UniprotKB					Best Blastp Match NCBI				
				Name	Accession number	Species	Evalue	Identity (%)	Name	Accession number	Species	Evalue	Identity (%)
**AludOR10a**	495	C	7	OR10a	A0A6M9TZ65	*Bactrocera dorsalis*	0	71.27	unnamed	CAD7012964	*Ceratitis capitata*	0	71.84
**AludOR13a**	450	C	6	OR13a1	A0A6M9TZJ2	*Zeugodacus tau*	0	76.3	OR13a-like	XP036345084	*Rhagoletis pomonella*	0	79.78
**AludOR22c**	398	C	6	LOC105230821	A0A6I9VI76	*Bactrocera dorsalis*	0	67.5	OR22c	XP036328669	*Rhagoletis pomonella*	0	71.57
**AludOR24a**	397	C	7	LOC105223483	A0A6I9UW31	*Bactrocera dorsalis*	0	73.55	OR24a-like	XP036347045	*Rhagoletis pomonella*	0	74.62
**AludOR2a**	357	C	4	OR19a	A0A6M9TYG5	*Zeugodacus cucurbitae*	0	76.11	OR2a-like	XP039962547	*Bactrocera tryoni*	0	76.4
**AludOR30a-like1**	287	3p	5	BlatOR30a	A0A5H2X5F2	*Bactrocera latifrons*	4.59E-60	38.32	OR2-like	XP036342137	*Rhagoletis pomonella*	2.08E-114	76.47
**AludOR30a-like2**	377	C	5	BlatOR30a	A0A5H2X5F2	*Bactrocera latifrons*	3.97E-81	37.63	unnamed	CAD6993154	*Ceratitis capitata*	2.05E-101	38.87
**AludOR33ab1**	378	C	6	OR33ab2	A0A3G2LEJ3	*Bactrocera minax*	0	81.38	unnamed	CAD6998182	*Ceratitis capitata*	0	80.69
**AludOR33ab2**	390	C	5	OR33ab1-1	A0A3G2LEI7	*Bactrocera minax*	0	76.86	OR33b-like	XP036329522	*Rhagoletis pomonella*	0	81.49
**AludOR42a**	419	C	6	OR42a	A0A6M9TZI9	*Zeugodacus tau*	0	65.12	OR7a-like	XP039959863	*Bactrocera tryoni*	0	63.47
**AludOR43a3**	369	C	6	ZcucOR43a-3	A0A5H2WW15	*Zeugodacus cucurbitae*	7.08E-152	57.07	OR2-like	XP036328946	*Rhagoletis pomonella*	0	65.16
**AludOR45a**	390	C	5	LOC105232464	A0A6I9VKB7	*Bactrocera dorsalis*	0	76.41	OR45a-like	XP036345921	*Rhagoletis pomonella*	0	78.46
**AludOR47b**	452	C	7	OR47b	A0A6M9TZ74	*Bactrocera dorsalis*	0	72.89	OR47b	XP039969307	*Bactrocera tryoni*	0	72.67
**AludOR49a**	397	C	6	OR49a	A0A6M9TYG4	*Bactrocera correcta*	0	82.7	OR49a-like	XP039950622	*Bactrocera tryoni*	0	82.7
**AludOR49b**	394	C	6	OR49b-1	A0A0G2UEX4	*Bactrocera dorsalis*	0	90.3	OR49b	XP036330492	*Rhagoletis pomonella*	0	91.91
**AludOR5**	385	C	7	OR5-1	A0A3G2LEM6	*Bactrocera minax*	0	75.33	OR33b-like	XP036336914	*Rhagoletis pomonella*	0	81.3

Gene	ORF (aa)	Type ORF	TMH	Best Blastp Match UniprotKB					Best Blastp Match NCBI				
				Name	Accession number	Species	Evalue	Identity (%)	Name	Accession number	Species	Evalue	Identity (%)
**AludOR59a-like**	365	5p	6	OR59a	A0A6I9VFX8	*Bactrocera dorsalis*	3.22E-95	66.96	OR59a-like	XP039957889	*Bactrocera tryoni*	0	68.49
**AludOR59a1**	392	C	5	OR59a.1	A0A6M9TYW4	*Bactrocera dorsalis*	0	77.78	OR59a-like	XP036332512	*Rhagoletis pomonella*	0	84.95
**AludOR59b-like**	457	C	6	OR59B	W8C1I4	*Ceratitis capitata*	6.85E-166	76.6	OR7a-like	XP036342571	*Rhagoletis pomonella*	0	84.1
**AludOR63a**	380	C	7	LOC105225172	A0A6I9VEW0	*Bactrocera dorsalis*	0	73.01	OR63a-like	XP036336255	*Rhagoletis pomonella*	0	80.26
**AludOR63a-like1**	413	C	7	OR63a-1	A0A0G2UMW5	*Bactrocera dorsalis*	0	64.98	OR63a-like	XP039963175	*Bactrocera tryoni*	0	65.46
**AludOR63a-like2**	417	C	5	OR63a.3	A0A6M9TZ99	*Bactrocera dorsalis*	0	73.14	OR63a-like	XP036336885	*Rhagoletis pomonella*	0	76.98
**AludOR67c**	413	C	6	BlatOR67c-v1	A0A5H2X5I6	*Bactrocera latifrons*	0	61.44	OR67c-like	XP036327494	*Rhagoletis pomonella*	0	75.19
**AludOR67d1**	388	C	6	BlatOR67d-3-v1	A0A5H2X9Q8	*Bactrocera latifrons*	0	76.23	OR67d-like	XP036342589	*Rhagoletis pomonella*	0	77.02
**AludOR67d2**	394	C	6	ZcucOR67d-1-v1	A0A5H2WZD3	*Zeugodacus cucurbitae*	0	70.62	OR67d-like	XP036336897	*Rhagoletis pomonella*	0	79.9
**AludOR69a1**	413	C	6	OR69a.1	A0A6M9TZU3	*Zeugodacus tau*	0	73.91	OR69a	XP036327301	*Rhagoletis pomonella*	0	78.94
**AludOR69a2**	425	C	6	OR69a.2	A0A6M9TZP3	*Zeugodacus tau*	5.94E-175	55.66	OR69a	XP036327296	*Rhagoletis pomonella*	0	66.75
**AludOR71a1**	354	5p	4	OR94b	A0A0L0BUZ0	*Lucilia cuprina*	4.79E-106	43.35	OR94a-like	XP036332422	*Rhagoletis pomonella*	6.97E-136	53.26
**AludOR71a2**	388	5p	7	OR94b	A0A0L0BUZ0	*Lucilia cuprina*	1.10E-114	45.29	OR94a-like	XP036332337	*Rhagoletis pomonella*	0	70.47
**AludOR74a**	402	C	5	OR74a1	A0A3G2LEK3	*Bactrocera minax*	0	68.31	OR74a-like	XP036329321	*Rhagoletis pomonella*	0	74.81
**AludOR74a-like1**	402	C	7	OR	A0A1I8M0X2	*Musca domestica*	6.71E-87	36.07	OR74a-like	XP036337022	*Rhagoletis pomonella*	1.55E-139	72.8
**AludOR74a2**	415	C	5	OR74a	A0A6M9TZI7	*Bactrocera dorsalis*	0	71.74	OR74a-like	XP036344413	*Rhagoletis pomonella*	0	75.42
**AludOR7a6**	218	3p	4	BdorOR42b	A0A348AZQ4	*Bactrocera*	6.68E-107	70.19	OR42b-like	XP036325260	*Rhagoletis*	2.16E-117	82.78

Gene	ORF (aa)	Type ORF	TMH	Best Blastp Match UniprotKB					Best Blastp Match NCBI				
				Name	Accession number	Species	Evalue	Identity (%)	Name	Accession number	Species	Evalue	Identity (%)
						dorsalis					*pomonella*		
**AludOR7a7**	431	C	5	OR7a.7	A0A6M9TZ91	*Bactrocera dorsalis*	0	80.32	OR7a-like	XP039960759	*Bactrocera tryoni*	0	80.05
**AludOR7a8**	419	C	6	OR42a	A0A6M9TZI9	*Zeugodacus tau*	0	71.24	OR7a-like	XP039959863	*Bactrocera tryoni*	0	69.35
**AludOR82a**	394	C	6	OR82a	A0A6M9TZ86	*Zeugodacus cucurbitae*	0	86.18	OR82a	XP036325270	*Rhagoletis pomonella*	0	85.35
**AludOR83a1**	476	5p	7	LOC105226603	A0A6I9VJU7	*Bactrocera dorsalis*	6.11E-163	51.55	OR83a-like	XP036229332	*Bactrocera oleae*	0	57.82
**AludOR85c**	410	C	6	LOC105230489	A0A6I9VAM7	*Bactrocera dorsalis*	1.55E-166	54.96	unnamed	CAD6996158	*Ceratitis capitata*	3.08E-173	56.76
**AludOR85d2**	110	5p	1	OR85d2	A0A3G2LEL9	*Bactrocera minax*	4.16E-55	76.15	OR85d	XP039948769	*Bactrocera tryoni*	2.32E-56	77.06
**AludOR88a**	410	C	2	OR88a	A0A6M9U071	*Zeugodacus tau*	0	63.77	OR88a	XP036332705	*Rhagoletis pomonella*	0	72.99
**AludOR94b2**	407	C	6	LOC105224900	A0A6J0RKF7	*Bactrocera dorsalis*	0	75.98	OR94a-like	XP039966652	*Bactrocera tryoni*	0	75.49
**AludORCO**	474	C	7	ORCO	A0A7H9SRD3	*Procecidochare s utilis*	0	96.19	ORCO	XP039970704	*Bactrocera tryoni*	0	95.77

ORF: Open Reading Frame, C: complete, 5p: 5prime partial, I: internal partial; THMM: transmembrane helices in proteins

Table S7. Best Blastp match of *A. ludens* candidate GRs

Gene	ORF (aa)	Type ORF	TMH	Best Blastp Match UniprotKB					Best Blastp Match NCBI				
				Name	Accession number	Species	Evalue	Identity (%)	Name	Accession number	Species	Evalue	Identity (%)
**AludGR21a**	450	C	6	GR21a	A0A6M9TZ08	*Bactrocera dorsalis*	0	96.44	GR21a-like	XP039957155	*Bactrocera tryoni*	0	96.22
**AludGR22**	405	C	8	GR22	W8CBK6	*Ceratitis capitata*	0	82.21	GR22-like	XP036221660	*Bactrocera oleae*	0	81.2
**AludGR22e**	138	5p	2	LOC105231253	A0A6I9VKH2	*Bactrocera dorsalis*	8.63E-57	67.69	GR22e-like	XP036329741	*Rhagoletis pomonella*	1.10E-59	68.12

Gene	ORF (aa)	Type ORF	TMH	Best Blastp Match UniprotKB					Best Blastp Match NCBI				
				Name	Accession number	Species	Evalue	Identity (%)	Name	Accession number	Species	Evalue	Identity (%)
**AludGR28a**	256	I	4	LOC105223070	A0A6I9V844	*Bactrocera dorsalis*	2.66E-147	87.31	GR28a	XP036328377	*Rhagoletis pomonella*	8.02E-156	90.77
**AludGR28b**	465	C	7	Gr28b	A0A0K8WM41	*Bactrocera latifrons*	0	82.04	GR28b	XP036214738	*Bactrocera oleae*	0	83.23
**AludGR2a**	416	C	7	LOC105228627	A0A6I9V595	*Bactrocera dorsalis*	0	67.8	GR2a	XP036338634	*Rhagoletis pomonella*	0	72.49
**AludGR33a**	500	C	8	Dana\GF14188	B3MNV7	*Drosophila ananassae*	5.48E-179	52.75	GR33a	XP039952326	*Bactrocera tryoni*	0	70.71
**AludGR39b**	122	5p	1	LOC105228630	A0A6I9V9T1	*Bactrocera dorsalis*	3.11E-24	60.26	GR39b	XP039961112	*Bactrocera tryoni*	9.04E-30	58.51
**AludGR43a**	320	I	5	Gr43a	A0A0B4LEE5	*Drosophila melanogaster*	1.44E-138	60.12	GR43a	XP039955593	*Bactrocera tryoni*	0	84.11
**AludGR5a**	172	5p	3	GR5a	A0A6M9TZC4	*Bactrocera dorsalis*	1.29E-108	87.79	GR5a	XP039965716	*Bactrocera tryoni*	1.11E-108	88.95
**AludGR63a**	483	C	7	BdorGR63a	A0A348AZV9	*Bactrocera dorsalis*	0	89.9	GR63a	XP039964488	*Bactrocera tryoni*	0	89.28
**AludGR64a**	396	5p	7	LOC105233094	A0A6I9W8J0	*Bactrocera dorsalis*	0	80.98	GR64a-like	XP039964231	*Bactrocera tryoni*	0	80.15
**AludGR64b1**	217	5p	4	BlatGR64b	A0A5H2X5P1	*Bactrocera latifrons*	2.67E-137	84.82	GR64b	XP036220169	*Bactrocera oleae*	9.49E-142	87.56
**AludGR64b2**	219	I	3	BlatGR64b	A0A5H2X5P1	*Bactrocera latifrons*	2.11E-98	77.78	GR64b	XP036337006	*Rhagoletis pomonella*	1.45E-106	71.06
**AludGR64c**	420	5p	7	LOC105233096	A0A6I9VR07	*Bactrocera dorsalis*	0	82.14	GR64c-like	XP039964232	*Bactrocera tryoni*	0	81.67
**AludGR64e**	468	C	6	GR64e	A0A6M9TYX0	*Zeugodacus cucurbitae*	0	86.33	GR64b-like	XP039964233	*Bactrocera tryoni*	0	83.38
**AludGR64f**	513	C	8	GR64f	A0A6M9TYJ9	*Bactrocera correcta*	0	80.51	GR64f-like	XP039965713	*Bactrocera tryoni*	0	80.51
**AludGR66a**	524	C	6	GR	A0A7H9SR73	*Procecidochares utilis*	0	75.67	GR66a	XP036338271	*Rhagoletis pomonella*	0	81.5
**AludGR68a**	233	5p	3	GR32a.2	A0A6M9TZD6	*Bactrocera dorsalis*	4.82E-101	63.2	GR32a-like	XP036328013	*Rhagoletis pomonella*	3.48E-102	64.94
**AludGR8a**	395	3p	8	GR08A	W8CDW6	*Ceratitis*	9.17E-167	70.12	GR8a-like	XP036222468	*Bactrocera oleae*	0	66.32

Gene	ORF (aa)	Type ORF	TMH	Best Blastp Match UniprotKB					Best Blastp Match NCBI				
				Name	Accession number	Species	Evalue	Identity (%)	Name	Accession number	Species	Evalue	Identity (%)
						capitata							
**AludGR94a**	397	C	5	GR94A	W8BZD0	*Ceratitis capitata*	0	63.41	GR94a	XP036224220	*Bactrocera oleae*	0	66.92
**AludGR98a**	342	3p	7	Dmoj\GI24562	B4K4J9	*Drosophila mojavensis*	1.10E-21	26.17	GR98a	XP036325256	*Rhagoletis pomonella*	1.37E-146	58.84
**AludGR98b**	196	5p	3	LOC109579632	A0A6J0RMD9	*Bactrocera dorsalis*	1.76E-103	75.51	G98b	XP036325255	*Rhagoletis pomonella*	6.60E-118	86.24

ORF: Open Reading Frame, C: complete, 5p: 5prime partial, I: internal partial; THMM: transmembrane helices in proteins

Table S8. Best Blastp match of *A. ludens* candidate IRs

Gene	ORF (aa)	Type ORF	TMH	Best Blastp Match UniprotKB					Best Blastp Match NCBI				
				Name	Accession number	Species	Evalue	Identity (%)	Name	Accession number	Species	Evalue	Identity (%)
**AludIR100a**	392	I	2	LOC118737125	XP036323319	*Rhagoletis pomonella*	0	80.41	LOC105231923	A0A6J0RQF6	*Bactrocera dorsalis*	0	76.98
**AludIR21a**	425	I	4	IR21a	XP011210105	*Bactrocera dorsalis*	0	91.06	IR21a	A0A6I9VG03	*Bactrocera dorsalis*	0	91.11
**AludIR25a**	940	C	3	IR25a	XP039951036	*Bactrocera tryoni*	0	95.75	GRIK2	A0A034WU95	*Bactrocera dorsalis*	0	95.64
**AludIR40a**	815	C	3	IR40a	XP039952224	*Bactrocera tryoni*	0	88.83	IR40a	A0A6M9TY60	*Bactrocera correcta*	0	89.73
**AludIR41a**	674	C	5	LOC120772140	XP039956500	*Bactrocera tryoni*	0	80.27	IR41a	A0A6M9TZ93	*Zeugodacus cucurbitae*	0	80.86
**AludIR56c**	183	I	1	unnamed	CAD7011917	*Ceratitis capitata*	0	73.51	BdorIR56c	A0A348AZX9	*Bactrocera dorsalis*	0	79.01
**AludIR31a**	566	5p	2	IR75a	XP039969934	*Bactrocera tryoni*	0	60.27	LOC115631400	A0A6J2U9W0	*Drosophila lebanonensis*	0	49.23
**AludIR75a-2**	543	I	3	IR75a-like	XP036327584	*Rhagoletis pomonella*	0	73.84	IR75a2	A0A6M9U0I3	*Zeugodacus tau*	0	65.86
**AludIR75a-1**	333	I	3	IR75a-like	XP036327349	*Rhagoletis pomonella*	0	90.12	IR75a1	A0A6M9TZ16	*Zeugodacus cucurbitae*	0	91.32
**AludIR75d**	713	C	4	LOC120778042	XP039965651	*Bactrocera tryoni*	0	78.63	IR75d	A0A6M9TZX6	*Zeugodacus tau*	0	78.26
**AludIR76a**	672	C	1	LOC118740053	XP036327442	*Rhagoletis pomonella*	0	85.42	IR76a	A0A5H2WWJ3	*Bactrocera latifrons*	0	83.99

Gene	ORF (aa)	Type ORF	TMH	Best Blastp Match UniprotKB					Best Blastp Match NCBI				
				Name	Accession number	Species	Evalue	Identity (%)	Name	Accession number	Species	Evalu e	Identity (%)
**AludIR8a**	952	C	3	IR25a	XP036324538	*Rhagoletis pomonella*	0	85.27	IR8a	A0A5H2X122	*Zeugodacus cucurbitae*	0	84.19
**AludIR92a**	718	C	2	LOC120766544	XP039948057	*Bactrocera tryoni*	0	70	IR92a	A0A6M9TZR6	*Zeugodacus tau*	0	70.33
**AludIR93a**	866	C	4	IR93a	XP036323735	*Rhagoletis pomonella*	0	85.22	IGRdelta1	W8BGJ6	*Ceratitis capitata*	0	83.82
**AludIR7c**	626	C	3	IR-delta	XP036320037	*Rhagoletis pomonella*	0	73.84	Lig_chan-Glu	A0A1I8MBZ8	*Musca domestica*	0	40.35
**AludKaiR1D**	875	3p	2	IGR-kainate2 iso2	XP036228939	*Bactrocera oleae*	0	80.81	IGR-kainate2	A0A6M9TYN1	*Zeugodacus cucurbitae*	0	80.33
**AludGluIID**	900	C	3	IGR-kainate2 iso1	XP039970451	*Bactrocera tryoni*	0	79.13	IGR-kainate2	A0A140CQB9	*Zeugodacus cucurbitae*	0	78.97

ORF: Open Reading Frame, C: complete, 5p: 5prime partial, I: internal partial; THMM: transmembrane helices in proteins}

Table S9. Accession numbers of no receptor chemosensory proteins used in phylogenetic analysis

**OBPs**				**CSPs**		**SNMPs**	
Accession number	Name	Accession number	Name	Accession number	Name	Accession number	Name
A0A1D8QLL2	AfraOBP19a	W8C4V0	CcapOBP19A	A0A6M9TYW3	BcorCSP1	Q7QC49	AgamSNMP1
A0A1D8QLN3	AfraOBP19b	W8AGN5	CcapOBP56A	A0A6M9TYH9	BcorCSP2	Q7Q6R1	AgamSNMP2
A0A1D8QLL9	AfraOBP19c	W8BS14	CcapOBP56A	A0A6M9TYN6	BcorCSP3	A0A6M9TYM0	BcorSNMP1a
A0A0Y0PEE3	AfraOBP19d-like	W8CBW7	CcapOBP56D	A0A6M9TYT2	BcorCSP4	A0A6M9TY95	BcorSNMP1b
A0A1D8QLL8	AfraOBP47b	W8BU34	CcapOBP56H	B2D0J0	BdorCSP	A0A6M9TY93	BcorSNMP2
A0A1D8QLL5	AfraOBP49a	W8C0X8	CcapOBP56H	A0A6M9TZI3	BdorCSP3	A0A0G2UGL0	BdorSNMP1
A0A1D8QLN5	AfraOBP50a	W8C879	CcapOBP57C	A0A034WWI9	BdorPEBIII	A0A6M9TZL3	BdorSNMP1b
A0A1D8QLM1	AfraOBP50e	W8BYY3	CcapOBP99A	A0A034W0K7	BdorPEBIII	A0A034WBN1	BdorSNMP2
A0A0Y0PI40	AfraOBP56a-like	W8CD84	CcapOBP99A	A0A3G2LEE9	BminCSP2	A0A0K8V8V4	BlatSNMP1
A0A1D8QLM3	AfraOBP56d	W8C4C0	CcapOBP99A	QOC63332	BminCSP3	A0A0K8VL93	BlatSNMP2
A0A1D8QLM0	AfraOBP56h	W8BAM5	CcapOBP99A	A0A3G2LEF3	BminCSP3	W8BDX2	CcapSNMP1
A0A1D8QLM2	AfraOBP56h	W8BUK4	CcapOBP99A	A0A3G2LEF5	BminCSP4	W8C9T0	CcapSNMP1
A0A1D8QLM5	AfraOBP57c	W8ASY6	CcapOBP99A	A0A0G2UKC9	BminCSP4	W8BAT2	CcapSNMP2

OBPs				CSPs		SNMPs	
Accession number	Name	Accession number	Name	Accession number	Name	Accession number	Name
A0A1D8QLM4	AfraOBP59a	W8CAL3	CcapOBP99A	XP018796406	U-BlatCSP	A0A0B4KGL9	DmelSNMP1
A0A1D8QLM6	AfraOBP83cd	E2DAN9	DmelOBP19a	XP018784824	U-BlatCSP	E1JI63	DmelSNMP2
A0A1D8QLM9	AfraOBP83ef	E2DBA6	DmelOBP19b	XP018793511	P-BlatPEBIII	A0A0A1XSX8	ZcucSNMP1
A0A1D8QLM7	AfraOBP83g	E2DC56	DmelOBP19c	XP014087037	U-BoleCSP3	A0A6M9TYQ5	ZcucSNMP1b
A0A1D8QLL7	AfraOBP8a	E2DCT2	DmelOBP19d	XP039962771	P-BtryA10	A0A0A1WYF3	ZcucSNMP2
A0A1D8QLM8	AfraOBP99a	P54195	DmelOBP28a	XP039954488	BtryPEBIII	A0A6M9TZQ5	ZtauSNMP1a
A0A0Y0P9P1	AfraOBP99a-like	Q7K084	DmelOBP44a	XP039957277	U-BtryCSP	A0A6M9TZW7	ZtauSNMP1b
A0A1D8QLN0	AfraOBP99b	A1Z875	DmelOBP46a	XP039962770	U-BtryCSP	A0A6M9TZZ2	ZtauSNMP2
A0A1D8QLN2	AfraOBP99c	A1Z8I9	DmelOBP47b	XP4529710	P-CcapA10		
A0A1D8QLN1	AfraOBP99d	A1Z9Q6	DmelOBP50e	XP012158387	CcapPEBIII		
A0A1D8QLH6	AoblOBP19a	Q8SY61	DmelOBP56d	XP004536817	U-CcapCSP		
A0A1D8QLJ0	AoblOBP19b	A9QI48	DmelOBP56g	XP004529711	U-CcapCSP		
A0A1D8QLJ9	AoblOBP19c	Q9V8Y9	DmelOBP56h	NP524121	DmelA10		
A0A1D8QLI5	AoblOBP47b	Q9V931	DmelOBP57c	NP001286808	DmelA10/PEBIII		
A0A1D8QLH9	AoblOBP49a	Q9W207	DmelOBP58d	NP001286870	U-DmelA10/PEBIII		
A0A1D8QLJ8	AoblOBP49a	Q86BF9	DmelOBP59a	AAA21358	DmelOSD		
A0A1D8QLI9	AoblOBP50a	Q7KUQ3	DmelOBP73a	NP001286811	U-DmelCG30172_A10		
A0A1D8QLI2	AoblOBP50e	P54193	DmelOBP83a	AAN71635	DmelRH70879p_A10		
A0A1L2JIV6	AoblOBP56a	Q9VNL1	DmelOBP83cd	QNL15632	U-PutiCSP		
A0A1D8QLK4	AoblOBP56d	P54194	DmelOBP84a	QNL15628	U-PutiCSP		
A0A1D8QLK2	AoblOBP56h	D1FYI8	DmelOBP99a	QNL15630	U-PutiCSP		
A0A1D8QLK0	AoblOBP56h	D1FYT5	DmelOBP99b	XP036319258	P-RpomA10		
A0A1D8QLK8	AoblOBP57c	A9QJL7	DmelOBP99c	XP17473899	U-RzepCSP		
A0A1D8QLK1	AoblOBP59a	D1FZL5	DmelOBP99d	XP011177222	U-ZcucCSP		
A0A1D8QLK3	AoblOBP83cd	A0A0A1XJZ1	ZcucOBP19a_0	A0A6M9TZ15	ZcucCSP3		
A0A1D8QLK5	AoblOBP83ef	A0A0A1WDN1	ZcucOBP19a_1	A0A0A1X6Y2	ZcucPEBIII		
A0A1D8QLK9	AoblOBP83g	A0A0A1XCI4	ZcucOBP56a_2	XP028901610	ZcucPEBIII-isoX1		
A0A1D8QLF8	AoblOBP8a	A0A0A1X269	ZcucOBP56a_5	ALS40433	U-ZtauA10		

OBPs				CSPs		SNMPs	
Accession number	Name	Accession number	Name	Accession number	Name	Accession number	Name
A0A1D8QLK7	AoblOBP99a	A0A0A1WII5	ZcucOBP56d_0	A0A6M9TZS5	ZtauCSP1		
A0A1D8QLL4	AoblOBP99b	A0A0A1WU34	ZcucOBP56d_1	A0A6M9U0F9	ZtauCSP2		
A0A1D8QLL3	AoblOBP99c	A0A0A1XS85	ZcucOBP56h_0	A0A6M9U0Q4	ZtauCSP3		
A0A1D8QLL1	AoblOBP99d	A0A0A1XCT5	ZcucOBP56h_1	A0A6M9U045	ZtauCSP4		
A0A034WVR1	BdorOBP19A	A0A0A1WWF8	ZcucOBP57c				
A0A034WLF0	BdorOBP19A	A0A0A1XJG7	ZcucOBP99a_0				
A0A034WQW6	BdorOBP56A	A0A0A1XRC5	ZcucOBP99a_3				
A0A034WTD0	BdorOBP56D	A0A0A1X819	ZcucOBP99a_4				
A0A034WRT9	BdorOBP56H	A0A0A1XN26	ZcucOBP99a_5				
A0A034W3Z7	BdorOBP57C	A0A0A1WVQ0	ZcucOBP99a_6				
A0A034VAP1	BdorOBP99A	A0A0A1X1Z8	ZcucOBP99a_7				
A0A034VWI9	BdorOBP99A	A0A1D6Y6I2	ZtauOBP11				
A0A034WIQ9	BdorOBP99A	A0A1D6Y6H0	ZtauOBP13				
A0A6I9W3H9	BdorOBP19d	A0A1D6Y6J3	ZtauOBP14				
A0A6J0RFI4	BdorOBP56a	A0A1D6Y6H9	ZtauOBP15				
A0A6I9UKC5	BdorOBP56d	A0A1D6Y6K4	ZtauOBP16				
A0A6I9VG65	BdorOBP56h	A0A1D6Y6J4	ZtauOBP18				
S5R7I3	BdorOBP7	A0A1D6Y6J5	ZtauOBP19				
A0A6I9VL90	BdorOBP70	A0A1D6Y6I6	ZtauOBP20				
A0A0K8VTX5	BlatOBP19a_0	A0A1D6Y6J0	ZtauOBP21				
A0A0K8TYL8	BlatOBP19a_1	A0A1D6Y6J1	ZtauOBP22				
A0A0K8VJ10	BlatOBP56a_4	A0A1D6Y6K3	ZtauOBP24				
A0A0K8VU37	BlatOBP56d_0	A0A1D6Y6I9	ZtauOBP25				
A0A0K8V5Y3	BlatOBP56h_0	A0A172BZK8	ZtauOBP3				
A0A0K8W7T9	BlatOBP56h_1	A0A172BZK6	ZtauOBP4				
A0A0K8U7K1	BlatOBP57c_1	A0A172BZK9	ZtauOBP5				
A0A0K8WLW4	BlatOBP99a_1	A0A1D6Y6H3	ZtauOBP7				
A0A0K8VEE3	BlatOBP99a_2	A0A1D6Y6I5	ZtauOBP8				

OBPs				CSPs		SNMPs	
Accession number	Name	Accession number	Name	Accession number	Name	Accession number	Name
A0A0K8UV06	BlatOBP99a_3						
A0A0K8VWQ2	BlatOBP99a_4						
A0A0K8WLU2	BlatOBP99a_5						
A0A0K8VYN4	BlatOBP99a_7						

OBPs: Odorant Binding Proteins; CSPs: Chemosensory Proteins; SNMPs: Sensory Neuronal Membrane Proteins;

Table S10. Accession numbers of receptor chemosensory proteins used in phylogenetic analysis

ORs				GRs		IRs	
Accession number	Name	Accession number	Name	Accession number	Name	Accession number	Name
A0A6M9TY59	BcorOR10a	XP036342060	RpomOR30a_like	A0A6M9TY81	BcorGR21a	A0A6M9TY61	BcorIR21a
A0A6M9TYC1	BcorOR13a_1	XP036336876	RpomOR30a_like	A0A6M9TY82	BcorGR22	A0A6M9TYI4	BcorIR25a
A0A6M9TY20	BcorOR19a	XP036329676	RpomOR33b_like	A0A6M9TY84	BcorGR28bC	A0A6M9TY60	BcorIR40a
A0A6M9TY23	BcorOR42a	XP036329522	RpomOR33b_like	A0A6M9TZ03	BcorGR32a_2	A0A6M9TY63	BcorIR41a
A0A6M9TY22	BcorOR43a_1	XP036336914	RpomOR33b_like	A0A6M9TYS5	BcorGR63a	A0A6M9TY62	BcorIR64a
A0A6M9TYT7	BcorOR43a_4a	XP036348085	RpomOR42a_like_partial	A0A6M9TYD4	BcorGR64e	A0A6M9TYX8	BcorIR75a_1
A0A6M9TYK4	BcorOR45a	XP036325260	RpomOR42b_like	A0A6M9TYJ9	BcorGR64f	A0A6M9TZ04	BcorIR75a_2
A0A6M9TYD8	BcorOR47b	XP036332306	RpomOR43a_like	A0A6M9TZ08	BdorGR21a	A0A6M9TYP6	BcorIR75d
A0A6M9TYG4	BcorOR49a	XP036345921	RpomOR45a_like	A0A348AZU9	BdorGR21a_2_v1	A0A6M9TYA9	BcorIR76a
A0A6M9TY32	BcorOR49b	XP036347151	RpomOR47b_like_partial	A0A034V2V0	BdorGR28A	A0A6M9TYJ7	BcorIR84a
A0A6M9TY38	BcorOR59a_1	XP036322429	RpomOR49a	A0A348AZV3	BdorGR28b	A0A6M9TYG3	BcorIR8a
A0A6M9TY34	BcorOR59a_2	XP036330492	RpomOR49b	A0A0G2UEL7	BdorGR28b	A0A6M9TY74	BcorIR92a
A0A6M9TY36	BcorOR63a_1	XP036336799	RpomOR59a_like	A0A034WHT2	BdorGR32A	A0A6M9TY73	BcorIR93a
A0A6M9TYY7	BcorOR63a_3	XP036332437	RpomOR59a_like	A0A034UY90	BdorGR33A	A0A0G2UGG7	BdorGluIID
A0A6M9TYL8	BcorOR67c_1	XP036332512	RpomOR59a_like	A0A348AZV6	BdorGR39b	A0A0G2UEM7	BdorIR100a
A0A6M9TYE9	BcorOR67d_2	XP036342619	RpomOR59b_like	A0A6M9TZ73	BdorGR5a	A0A6M9TZA0	BdorIR21a
A0A6M9TY45	BcorOR67d_4	XP036336886	RpomOR63a_like	A0A348AZV9	BdorGR63a	A0A348AZW9	BdorIR25a
A0A6M9TY48	BcorOR69a_1	XP036336885	RpomOR63a_like	A0A348AZW0	BdorGR64b	A0A348AZX1	BdorIR31a_2

ORs				GRs		IRs	
Accession number	Name	Accession number	Name	Accession number	Name	Accession number	Name
A0A6M9TY54	BcorOR69a_2	XP036336255	RpomOR63a_like	A0A6M9TZ21	BdorGR64e	A0A0G2UGF8	BdorIR40a
A0A6M9TY50	BcorOR74a	XP036327280	RpomOR67c_like	A0A6M9TZP5	BdorGR64f	A0A0G2UEM3	BdorIR41a
A0A6M9TYT0	BcorOR7a_6	XP036320221	RpomOR67c_like	A0A0G2UES1	BdorGR68a	A0A348AZX9	BdorIR56c_part2
A0A6M9TYW8	BcorOR7a_7	XP036325007	RpomOR67d_like	A0A348AZW5	BdorGR98b	A0A6M9TZR5	BdorIR64a
A0A6M9TYJ4	BcorOR7a_8	XP036337020	RpomOR67d_like	A0A5H2WZ00	BlatGR21a_1	A0A6M9TZB8	BdorIR75a_1
A0A6M9TYW5	BcorOR82a	XP036345777	RpomOR69a	A0A5H2X9T5	BlatGR21a_2_v1	A0A6M9TZ05	BdorIR75a_2
A0A6M9TYZ7	BcorOR88a	XP036327298	RpomOR69a	A0A0K8WM41	BlatGr28b	A0A6M9TZA5	BdorIR75d
A0A6M9TY89	BcorOR94b_1	XP036329321	RpomOR74a_like	A0A5H2X0N4	BlatGR28b_3	A0A6M9TZA1	BdorIR76a
A0A6M9TYE0	BcorOrco	XP036337022	RpomOR74a_like	A0A5H2WWF0	BlatGR32a_3	A0A0G2UES7	BdorIR84a
A0A0G2UKH1	BdorOR13a	XP036344417	RpomOR74a_like	A0A5H2WZ13	BlatGR33a	A0A348AZW8	BdorIR8a_2
A0A6M9TYV4	BdorOR19a	XP036332692	RpomOR7a_like	A0A5H2X9U7	BlatGR39b	A0A0G2UGG3	BdorIR92a
A0A034WVI2	BdorOR22C	XP036343945	RpomOR7a_like	A0A5H2X4L7	BlatGR43a_1_part1	A0A6M9TZI6	BdorIR93a
A0A0G2UGJ8	BdorOR43a_1	XP036325270	RpomOR82a	A0A5H2X034	BlatGR43a_1_part2	A0A5H2WVU9	BlatIR21a_part1
A0A6M9TZ79	BdorOR43a_4b	XP036342149	RpomOR83a	A0A5H2X0M0	BlatGR5a_3	A0A5H2X5R2	BlatIR25a
A0A0G2UKH6	BdorOR45a	XP036326587	RpomOR85c_like	A0A5H2WXV9	BlatGR63a	A0A5H2X9Z8	BlatIR41a_v2
A0A348AZR1	BdorOR47b	XP036344098	RpomOR85c_like_partial	A0A5H2X0P4	BlatGR64a	A0A5H2X081	BlatIR64a_1
A0A6M9TYX1	BdorOR49a	XP036326958	RpomOR85d	A0A5H2X5P1	BlatGR64b	A0A5H2X0R6	BlatIR75a_part2
A0A6M9TZK2	BdorOR49b	XP036332705	RpomOR88a	A0A5H2WVV8	BlatGR64c	A0A5H2X5S7	BlatIR75b
A0A6M9TYW4	BdorOR59a_1	XP036323149	RpomOR94a	A0A5H2WZ23	BlatGR64e_part2	A0A5H2WVZ7	BlatIR75d
A0A6M9TYW9	BdorOR59a_2	XP036332423	RpomOR94a_like	A0A5H2X4M7	BlatGR64f	A0A5H2WWJ3	BlatIR76a
A0A6M9TZE8	BdorOR63a_1	XP036332422	RpomOR94a_like	A0A5H2X038	BlatGR66a_part1	A0A5H2XA15	BlatIR84a
A0A6M9TZA2	BdorOR63a_3	XP036335587	RpomORCO	A0A5H2WVT9	BlatGR66a_part2	A0A5H2X049	BlatIR8a
A0A6I9V829	BdorOR67c	XP036328946	RpomOROr2_like	A0A5H2WXW6	BlatGR68a	A0A5H2WXY9	BlatIR92a_2_part2
A0A6M9TZB1	BdorOR67c_1	XP017476238	RzepOR10a	A0A5H2X5L3	BlatGR8a_1_part1	A0A5H2X5U1	BlatIR93a_1_part2
A0A348AZS8	BdorOR67d_1	XP017466349	RzepOR13a_like	A0A5H2X5Q1	BlatGR94a	Q9V9T2	DmelIR100a
A0A6M9TZ83	BdorOR67d_2	XP017480983	RzepOR22c	W8CDW6	CcapGR08A	Q9VPI2	DmelIR21a
A0A0G2UET3	BdorOR69a	XP017483268	RzepOR24a	W8CE61	CcapGR21A	Q9VR32	DmelIR25a

ORs				GRs		IRs	
Accession number	Name	Accession number	Name	Accession number	Name	Accession number	Name
A0A6M9TZ90	BdorOR69a_2	XP017490054	RzepOR2a_like_partial	W8CBK6	CcapGR22	Q8IPB8	DmelIR31a
A0A6I9UZ81	BdorOR74a	XP017483288	RzepOR33b_like	W8C773	CcapGR32A	X2JAN0	DmelIR40a
A0A6M9TZG0	BdorOR74a	XP017490193	RzepOR33b_like	W8BZD0	CcapGR94A	A1Z6D6	DmelIR41a
A0A0G2UEW4	BdorOR7a_2	XP017486793	RzepOR33b_like	Q9VPT1	DmelGR21a	A1ZBM8	DmelIR56c
A0A6M9TZ82	BdorOR7a_6	XP017483527	RzepOR42b_like	P58951	DmelGR22a	Q9VRL4	DmelIR64a
A0A6M9TZ91	BdorOR7a_7	XP017479408	RzepOR43a	P84181	DmelGR22d	Q9VVL1	DmelIR75a
A0A6M9TZ69	BdorOR7a_8	XP017494799	RzepOR47a_like_partial	P58953	DmelGR22e	Q9VVU7	DmelIR75d
A0A348AZT7	BdorOR82a	XP017471040	RzepOR47b	Q9VM09	DmelGR28a	B7Z084	DmelIR76a
A0A6I9UZ10	BdorOR83a_like	XP017477474	RzepOR49a_partial	Q9VM08	DmelGR28b	Q9W3P2	DmelIR7c
A0A0G2UKI5	BdorOR88a	XP017475280	RzepOR49b_isoform_X2	E1JJC5	DmelGR2a	Q9VIA5	DmelIR84a
A0A348AZU2	BdorOR94b_1	XP017476444	RzepOR59a_like	Q9VKJ7	DmelGR32a	E9NA95	DmelIR8a
A0A6M9TZU6	BdorOR94b_2	XP017487642	RzepOR59a_like	P58960	DmelGR39b	Q9VDN3	DmelIR92a
A0A5H2WXQ9	BlatOR10a	XP017488345	RzepOR63a	Q0E9G8	DmelGR43a	Q9VDH6	DmelIR93a
A0A5H2X0I7	BlatOR13a	XP017484939	RzepOR63a_like	Q9W497	DmelGR5a	NP650925	DmelKainate-IGR1D
A0A5H2WZW7	BlatOR2a_v1	XP017487849	RzepOR67c_like	Q9VZL7	DmelGR63a	XP036341412	RpomGRIK2_like
A0A5H2X9M7	BlatOR42b	XP017461882	RzepOR67c_like_partial	D7F9T2	DmelGR64a	XP036341411	RpomGRIK2_like
A0A5H2X4G3	BlatOR43a_1_v1	XP017473047	RzepOR67d_like	D7F9V2	DmelGR64b	XP036342815	RpomGRIK2 likeIsoform X1
A0A5H2X4H2	BlatOR59a	XP017468648	RzepOR67d_like	D7F9X8	DmelGR64c	XP036342817	RpomGRIK2_like_isoform_X2
A0A5H2WXS8	BlatOR63a_2	XP017471579	RzepOR69a	Q9VZJ6	DmelGR64d	XP036329707	RpomIR21a
A0A5H2X0K6	BlatOR63a_3	XP017465320	RzepOR69a	P83296	DmelGR64e	XP036324538	RpomIR25a
A0A5H2WWC4	BlatOR67d_1	XP017490047	RzepOR74a	P83297	DmelGR64f	XP036327744	RpomIR25a
A0A5H2X9Q8	BlatOR67d_3_v1	XP017469217	RzepOR74a_like	D7FA04	DmelGR66a	XP036322623	RpomIR40a
A0A5H2WVQ3	BlatOR69a_1	XP017473159	RzepOR74a_like	Q9VTN0	DmelGR68a	XP036327586	RpomIR75a
A0A5H2WXT7	BlatOR69a_2	XP017461213	RzepOR7a_like	Q9W367	DmelGR8a	XP036327349	RpomIR75a_like
A0A5H2X0K9	BlatOR74a_1	XP017474398	RzepOR7a_like	D7FAK6	DmelGR94a	XP036323735	RpomIR93a
A0A5H2WXQ4	BlatOR7a_1	XP017493400	RzepOR7a_like	D7FAQ2	DmelGR98a	XP036320037	RpomIRdelta1
A0A5H2WVM4	BlatOR7a_4	XP017481537	RzepOR82a	D7FAW4	DmelGR98b	A0A6M9TZQ6	ZtauIR21a

ORs				GRs		IRs	
Accession number	Name	Accession number	Name	Accession number	Name	Accession number	Name
A0A5H2WVS1	BlatOR82a	XP017478187	RzepOR83a	XP036334006	RpomGR21a_like	A0A6M9TYJ3	ZtauIR21a
A0A5H2WYX0	BlatOR88a	XP017482230	RzepOR85b_like	XP036329742	RpomGR22d	A0A6M9TZV6	ZtauIR25a
A0A5H2X4E1	BlatORCO	XP017472634	RzepOR85d	XP036329741	RpomGR22e_like	A0A5H2WWB5	ZtauIR25a
A0A3G2LEL1	BminOR1_1	XP017469100	RzepOR88a	XP036328377	RpomGR28a	A0A5H2WWT0	ZtauIR31a
A0A3G2LEI5	BminOR10a	XP017464263	RzepOR94a_like	XP036328378	RpomGR28b	A0A6M9TZL7	ZtauIR40a
A0A3G2LEI3	BminOR22c	XP017485906	RzepOR94a_like	XP036329791	RpomGR28b	A0A6M9TYJ6	ZtauIR40a
A0A7D4V5V3	BminOR24	XP017470946	RzepORisoform_X1	XP036338634	RpomGR2a	A0A6M9TZM1	ZtauIR41a
A0A3G2LEI9	BminOR24a	XP017483505	RzepOROr2_like	XP036329340	RpomGR32a_isoform_X1	A0A6M9TZ93	ZtauIR41a
A0A3G2LEI4	BminOR2a	A0A6M9TYJ2	ZcucOR10a	XP036328013	RpomGR32a_like	A0A6M9TZK7	ZtauIR64a
A0A3G2LEI7	BminOR33ab1_1	A0A6M9TYQ4	ZcucOR13a_1	XP036340902	RpomGR33a_like	A0A6M9TZF2	ZtauIR64a
A0A3G2LEM2	BminOR4_1	A0A6M9TYG5	ZcucOR19a	XP036338635	RpomGR39b	A0A6M9U083	ZtauIR75a_1
A0A3G2LEJ6	BminOR43a	A0A6M9TYH0	ZcucOR22c	XP036334658	RpomGR43a_like	A0A6M9TZ16	ZtauIR75a_1
A0A3G2LEJ8	BminOR45a1	A0A6M9TYF3	ZcucOR42a	XP036337263	RpomGR5a	A0A6M9U0I3	ZtauIR75a_2
A0A3G2LEJ2	BminOR47b	A0A5H2XA80	ZcucOR42b	XP036337481	RpomGR63a	A0A6M9TYN7	ZtauIR75a_2
A0A3G2LEJ9	BminOR49a	A0A5H2X4U4	ZcucOR43a_1	XP036337008	RpomGR64a	A0A6M9TZX6	ZtauIR75d
A0A6N0A5L8	BminOR5	A0A5H2X0B3	ZcucOR43a_2	XP036337006	RpomGR64b	A0A6M9TYV6	ZtauIR75d
A0A3G2LEM6	BminOR5_1	A0A6M9TYK5	ZcucOR45a	XP036337005	RpomGR64c	A0A6M9TZN0	ZtauIR76a
A0A3G2LEM7	BminOR5_2	A0A5H2X0V3	ZcucOR47b_v1	XP036337010	RpomGR64e_like	A0A5H2X0I4	ZtauIR76a
A0A3G2LEJ7	BminOR63a1_1	A0A6M9TYH8	ZcucOR49a	XP036337262	RpomGR64f_like	A0A6M9TZV9	ZtauIR84a
A0A3G2LEK5	BminOR67c	A0A6M9TYI5	ZcucOR49b	XP036345248	RpomGR8a_like	A0A6M9TYM2	ZtauIR84a
A0A3G2LEK4	BminOR67d1	A0A6M9TYG9	ZcucOR59a_1	XP036323157	RpomGR94a_isoform_X1	A0A6M9TZU0	ZtauIR8a
A0A3G2LEK7	BminOR71a	A0A6M9TYG6	ZcucOR59a_2	XP036323158	RpomGR97a_isoform_X2	A0A6M9TYL1	ZtauIR8a
A0A3G2LEK9	BminOR74a2	A0A5H2X0W1	ZcucOR63a_2	XP036325256	RpomGR98a	A0A6M9TZR6	ZtauIR92a
A0A3G2LEI8	BminOR7a1	A0A6M9TYZ1	ZcucOR67c_1	XP036325255	RpomGR98b	A0A6M9TYK6	ZtauIR92a
A0A3G2LEI1	BminOR7a2	A0A6M9TYT5	ZcucOR67d_2	XP036338271	RpomGRfor66a	A0A6M9TZM7	ZtauIR93a
A0A3G2LEK6	BminOR82a	A0A6M9TYJ1	ZcucOR67d_4	A0A5H2WZF1	ZcucGR21a_1_v1	A0A6M9TYK7	ZtauIR93a
A0A3G2LEL5	BminOR83a1	A0A5H2X603	ZcucOR69a_1	A0A6M9TZB2	ZcucGR22		

ORs				GRs		IRs	
Accession number	Name	Accession number	Name	Accession number	Name	Accession number	Name
A0A3G2LEL9	BminOR85d2	A0A6M9TYI1	ZcucOR69a_2	A0A5H2X0E9	ZcucGR22d		
A0A3G2LEL4	BminOR88a	A0A5H2WWQ2	ZcucOR74a	A0A5H2WY49	ZcucGR28a		
A0A3G2LEL3	BminORCO	A0A5H2X094	ZcucOR7a_1	A0A5H2X110	ZcucGR28b		
W8C1I4	CcapOR59B	A0A6M9TZA7	ZcucOR7a_7	A0A6M9TZ35	ZcucGR32a_1		
W8BXK2	CcapOR83B	A0A6M9TZ86	ZcucOR82a	A0A6M9TYR0	ZcucGR63a		
W8AKN0	CcapOR85B	A0A6M9TZ06	ZcucOR88a	A0A5H2WZF8	ZcucGR64b_part1		
ADK48058	DemlOR7a	A0A6M9TYU6	ZcucOR94b_1	A0A5H2XAD8	ZcucGR64b_part2		
ADK48058	DemlOR7a	A0A6M9TYY3	ZcucOR94b_2	A0A6M9TYX0	ZcucGR64e		
E2E5B5	DmelOR10a	A0A0A1WPI9	ZcucOrco	A0A5H2X0F6	ZcucGR64f		
Q9VXL0	DmelOR13a	A0A6M9TZT8	ZtauOR10a	A0A5H2WWQ8	ZcucGR8a		
P81911	DmelOR22c	A0A6M9TZJ2	ZtauOR13a_1	A0A5H2WY56	ZcucGR94a		
P81913	DmelOR24a	A0A6M9TZT0	ZtauOR19a	A0A6M9TZN7	ZtauGR21a		
D3PK91	DmelOR2a_RA	A0A6M9TZL6	ZtauOR22c	A0A6M9TZN8	ZtauGR22		
P81914	DmelOR33a	A0A6M9TZI9	ZtauOR42a	A0A6M9U0B0	ZtauGR32a_1		
P81915	DmelOR33b	A0A6M9TZJ9	ZtauOR43a_1	A0A6M9U0L0	ZtauGR63a		
ADK48356	DmelOR43a	A0A6M9U0G5	ZtauOR43a_4	A0A6M9U014	ZtauGR64e		
Q9V568	DmelOR45a	A0A6M9TZU8	ZtauOR45a				
P81922	DmelOR47b	A0A6M9TZS1	ZtauOR47b				
Q9V6A9	DmelOR49a	A0A6M9TZT9	ZtauOR49a				
Q9V6H2	DmelOR49b	A0A6M9TZM9	ZtauOR49b				
NP523821	DmelOR59a	A0A6M9TZK1	ZtauOR59a_1				
E5AJF0	DmelOR63a	A0A6M9TZK5	ZtauOR59a_2				
D7F8L2	DmelOR67c	A0A6M9TZJ3	ZtauOR63a_1				
D7F8B2	DmelOR69a	A0A6M9U064	ZtauOR67c_1				
D7F8C7	DmelOR71a	A0A6M9TZV3	ZtauOR67d_2				
D7F8F4	DmelOR74a	A0A6M9TZT2	ZtauOR67d_4				
D7F8N0	DmelOR82a	A0A6M9TZU3	ZtauOR69a_1				

ORs				GRs		IRs	
Accession number	Name	Accession number	Name	Accession number	Name	Accession number	Name
D7F8U6	DmelOR83a	A0A6M9TZP3	ZtauOR69a_2				
D7F8X2	DmelOR83b	A0A6M9TZK8	ZtauOR74a				
D7F954	DmelOR85b	A0A6M9TZH6	ZtauOR7a_6				
D7F9R4	DmelOR85c	A0A6M9U033	ZtauOR7a_7				
D7F960	DmelOR85d	A0A6M9U0D8	ZtauOR7a_8				
D7F9D7	DmelOR88a	A0A6M9TZL4	ZtauOR82a				
D7F9J6	DmelOR94a	A0A6M9TZK0	ZtauOR85c				
XP036333231	RpomOR10a	A0A6M9U071	ZtauOR88a				
XP036345084	RpomOR13a_like	A0A6M9TZW3	ZtauOR94b_1				
XP036328669	RpomOR22c	A0A6M9TZL9	ZtauOR94b_2				
XP036347045	RpomOR24a_like	A0A6M9TZS0	ZtauOrco				
XP036345750	RpomOR2a_like						

ORs: Odorant Receptors; Grs: Gustatory Receptors; IRs: Ionotropic Receptors

Table S11. Evolutionary models chosen by the BIC criterion for phylogenetic analyses of the six chemosensory protein families.

**Chemosensory Protein Family**		**Evolutionary models**
**Non Receptor**	Odorant Binding Proteins	WAG+I+G4
	Chemosensory Proteins	LG+G4
	Sensory Neuronal Membrane Proteins	LG+I+G4
**Receptor**	Odorant Receptors	JTT+G4+F
	Gustatory Receptors	VT+G4+F
	Ionotrópico Receptors	VT+G4+F
